# Holographic direct pulsed laser writing of two-dimensional nanostructures†
†Electronic supplementary information (ESI) available. See DOI: 10.1039/c6ra22241b



**DOI:** 10.1039/c6ra22241b

**Published:** 2016-11-22

**Authors:** Bader AlQattan, Haider Butt, Aydin Sabouri, Ali K. Yetisen, Rajib Ahmed, Nasim Mahmoodi

**Affiliations:** a Nanotechnology Laboratory, School of Engineering, University of Birmingham, Birmingham B15 2TT, UK. Email: h.butt@bham.ac.uk; Tel: +44 (0)1214158623; b Harvard Medical School and Wellman Center for Photomedicine, Massachusetts General Hospital, 65 Landsdowne Street, Cambridge, MA 02139, USA; c Harvard-MIT Division of Health Sciences and Technology, Massachusetts Institute of Technology, Cambridge, MA 02139, USA

## Abstract

The development of accurate and rapid techniques to produce nanophotonic structures is essential in data storage, sensors, and spectroscopy. Existing bottom-up and top-down approaches to fabricate nanophotonic devices are high cost and time consuming, limiting their mass manufacturing and practical applications. Here, we demonstrate a strategy to rapidly create 25–40 nm thick 1/2D Au–Ti nanopatterns using holographic direct laser interference patterning (DLIP). Pulses of an Nd:YAG laser (1064 nm) in holographic Denisyuk reflection mode were used to create ablative interference fringes. The constructive interference antinode regions of the standing wave selectively ablated a Au–Ti layer in localized regions to controllably form nanogratings. Varying the laser exposure parameters allowed for rapid patterning of 2D square and rectangular arrays within seconds. Controlling the distances between the laser source, recording medium, and the object, allowed for achieving a 2D spatial grating periodicity of 640 nm × 640 nm. Diffracted and transmitted light spectra of 2D nanostructure arrays were analyzed using angle-resolved measurements and spectroscopy.

## Abstract 2

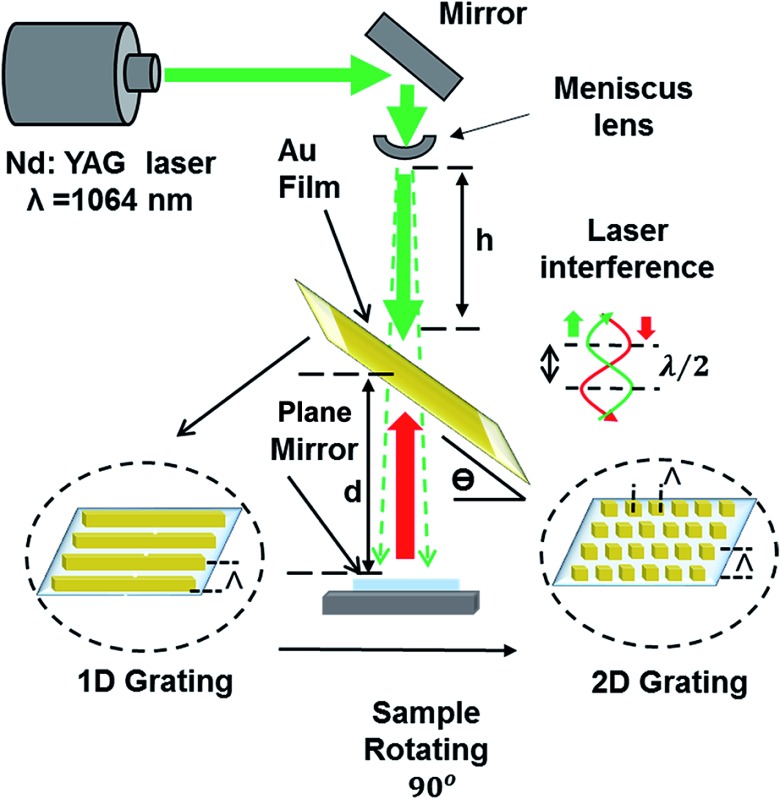
The development of accurate and rapid techniques to produce nanophotonic structures is essential in data storage, sensors, and spectroscopy.

## Introduction

Photonic materials and periodic nanostructures have a myriad of applications in sensing, spectroscopy, telecommunications, and security industries.^1–4^ Photonic structures can be fabricated by many advanced processes that may achieve resolution down to 10 nm; however, achieving precise and uniform structures requires sophisticated equipment and time consuming fabrication processes.^5,6^ Bottom-up fabrication of photonic crystals through self-assembly involves ion depletion of highly-charged particles from the solution requiring lengthy dialysis times.^7^ Similarly, top-down fabrication of nanophotonic devices through e-beam writing over 1 cm^2^ can take from hours to days to complete.^8^ Hence, there is a clear need to develop low-cost fabrication techniques to rapidly fabricate large area periodic nanostructures with high resolution. Laser ablation compared to lithography/e-beam milling for fabrication of micro/nano structures is known as a faster method, moreover, it requires less number of processing steps.^9,10^ Laser ablation has been utilized to create optical devices operating in the visible region.^11,12^ Direct laser writing procedures have low energy requirements and rapid production times not only for 1D but also for 2D nanostructures.^13,14^ In particular, creating 2D structural gratings with high-resolution is highly desirable for optical applications including lasing, biosensing, and spectroscopy.^15^ Likewise, direct laser interference patterning (DLIP), where single laser beam is split to produce interference of multiple laser beams on a target substrate, is capable of rapidly generating 2D nanopatterns. However, it has the disadvantages of requiring complex optical setups and precise control over system parameters, which increase the cost. Combining the advantages of DLIP with holographic DLIP can reduce the cost and time. The system involves an original laser beam reflection mirrors to allow rapid creation of high-resolution 1D and 2D surface gratings.^2,3^

We previously described a laser patterning technique to create 1D ink gratings using a holographic patterning system.^13^ The interference of laser light in a multilayer field allowed the ink to be ablated in antinode (constructive) regions of a recording medium.^2^ The utilization of this approach created 1D parallel and radial gratings.^3^ However, the effect of patterning parameters on the formation of surface gratings was not studied. In addition to the interference pattern geometry, understanding the mechanism and the effect of optical factors such as interference angle and superposition of the interference waves in the creation of gratings will allow controllable fabrication and optimization of the grating geometry. Also, the ability to rapidly fabricate surface gratings in 2D patterns remains elusive with this approach.

Here, we demonstrate a DLIP strategy for rapid fabrication of 2D nanostructures at low cost. A pulsed nanosecond (ns) laser in holographic Denisyuk reflection mode was used to produce gratings in various metal thin films deposited on glass substrates. The system utilized a highly-intense pulse, which created an interference pattern to ablate localized regions in the metal layer. We demonstrate this fabrication technique to tune the parameters in order to control the geometry and spatial periodicity of 1D and 2D gratings in squares and rectangular (parallelograms) arrays. Light diffraction from the 2D nanostructures was spectrally analyzed and angle-resolved measurements were performed to characterize the fabricated gratings.

## Results and discussion

### Fabrication technique

**Fig. 1:**
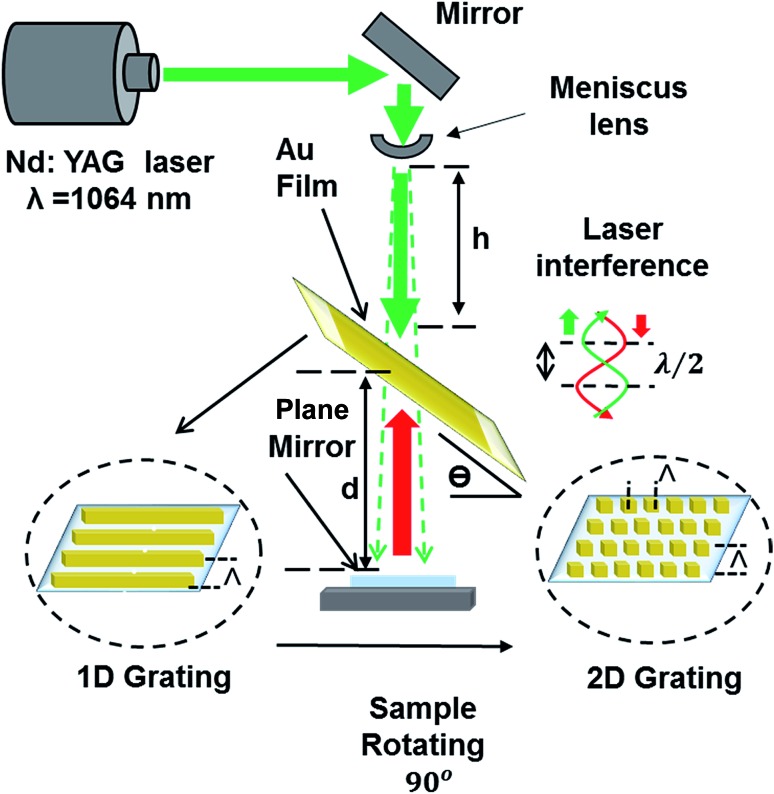
Fabrication of 1/2D patterns through nanosecond laser light interference in DLIP Denisyuk reflection mode. Nd:YAG laser beam (3.5 ns) propagates through a converging lens and reflected back from a plane mirror to ablate the localized regions of the recording medium (metal film).

Fig. 1 shows the schematic of the hologram recording setup in Denisyuk ablation mode. The laser reference beam initially passes through a meniscus converging lens to be focused (focal length ∼ 8.55 cm) then it defocuses to form diverging beam. The lens increases the size of beam after the focus point as it moves in the *z*-axis (vertical) and the ablated spot diameter in Au film expand. The focal point (*f*) of the lens is:1
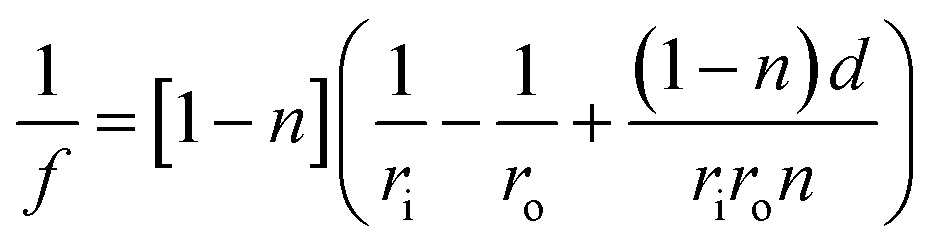
where *r*_i_ is the inner radius, *r*_o_ is the outer radius, *n* is the reflective index, and *d* is the lens thickness. The reference beam propagates through the thin metal film and is reflected back from the underlying mirror. The reflected laser beam (object beam) interferes with the reference beam to create concentrated energy zones (interference fringes) in horizontal and vertical directions, ablating the thin film at energy-concentrated antinode regions (constructive interference). The interference caused by the nodes of magnetic field overlap with antinodes of electric field. The electric (*E*) and magnetic (*H*) field vectors are:2
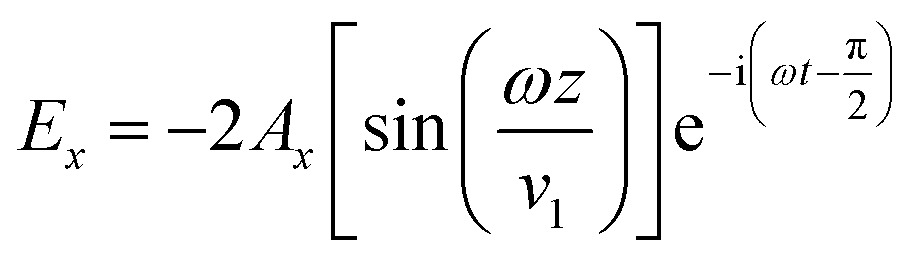

3
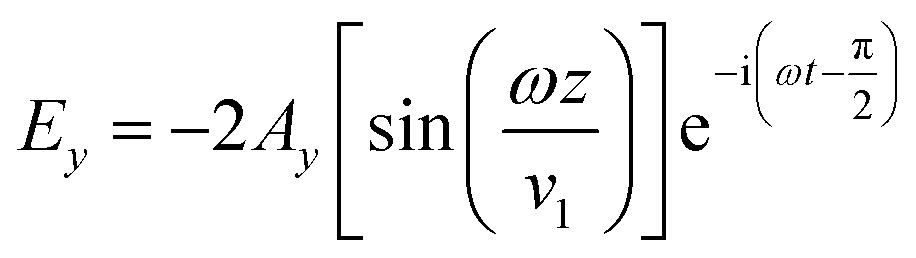

4 *E*_*z*_ = *H*_*z*_ = 0

5
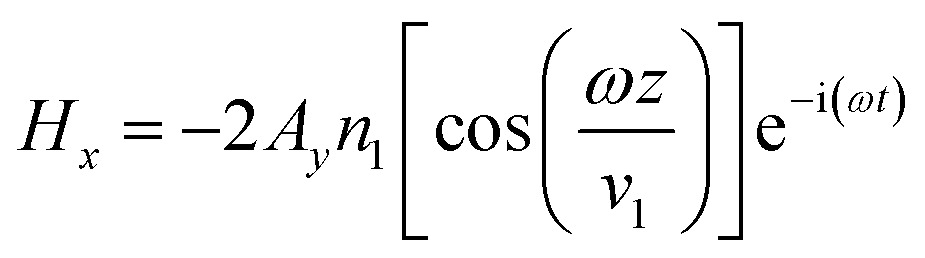

6
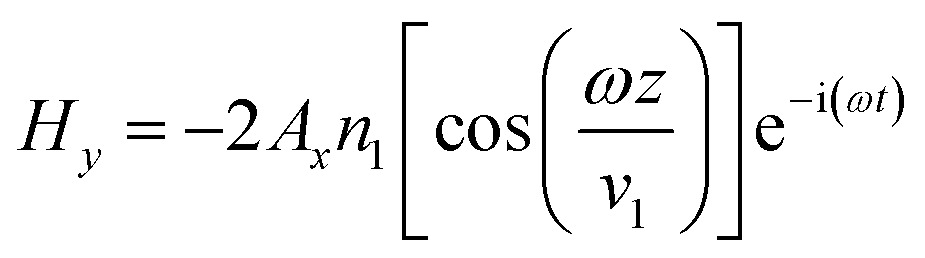
where *A*, *ω*, *t*, and *λ*_o_ represent axis plane, angular velocity, time, and wavelength, respectively. The position of antinodes can be predicted by 
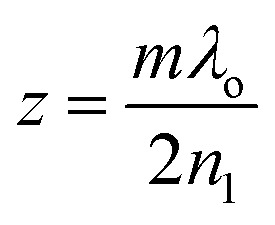
.^16^

On ablation of metal film, a circular well-ordered grating area of ∼1 cm diameter is produced (roughly the size of laser beam). In the present work, holographic DLIP was performed on Au thin films (25 and 40 nm thicknesses), which were deposited on Ti films (4 nm) acting as an adhesion layer.^17^ Holographic DLIP was performed at various incident angles, and experimental results were compared with the theory. In addition, effects of changing the laser beam-sample distance and sample-mirror distance on the grating spacing were evaluated. Au film spot size of the samples enlarged as the (*h*) distance was increased (ESI Material, Fig. S1†).

### Computational modeling of the focused beam interference

To understand the optical interference-induced patterning with 1064 nm focused beam (through biconvex lens), which produce a grating in the Au film, computational modeling was performed using COMSOL Multiphysics simulation package based finite element method (FEM).^18,19^ Scattering and periodic boundary conditions were set to define the interference pattern. The computational area was created with triangular meshing elements. The maximum degree of freedom was ∼126 884. Completed mesh consisted of 17 963 domain elements and 1183 boundary elements. Convergence test was carried out with fine mesh elements for improving the result accuracy.^20^

**Fig. 2:**
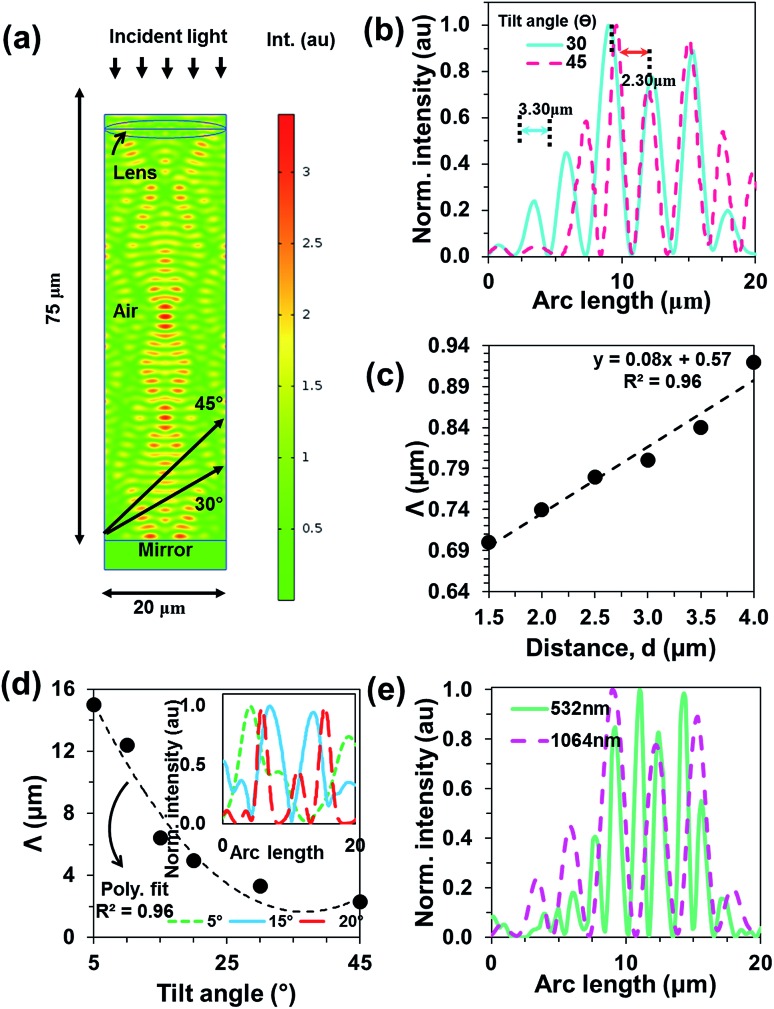
FEM modeling of the focused beam interference. (a) Computational geometry for the focused laser beam interference and its 2D intensity distribution. (b) Normalized intensity profile plotted for the focused laser light interference at planes of 30° and 45°, respectively. (c and d) Focused beam interference spacing as a function of distance between mirror and the observation plane (*d*) and tilt angle (*θ*). Inset shows normalized intensity profiles as a function of arc lengths. (e) Grating spacing as a function of wavelength.

**Fig. 3:**
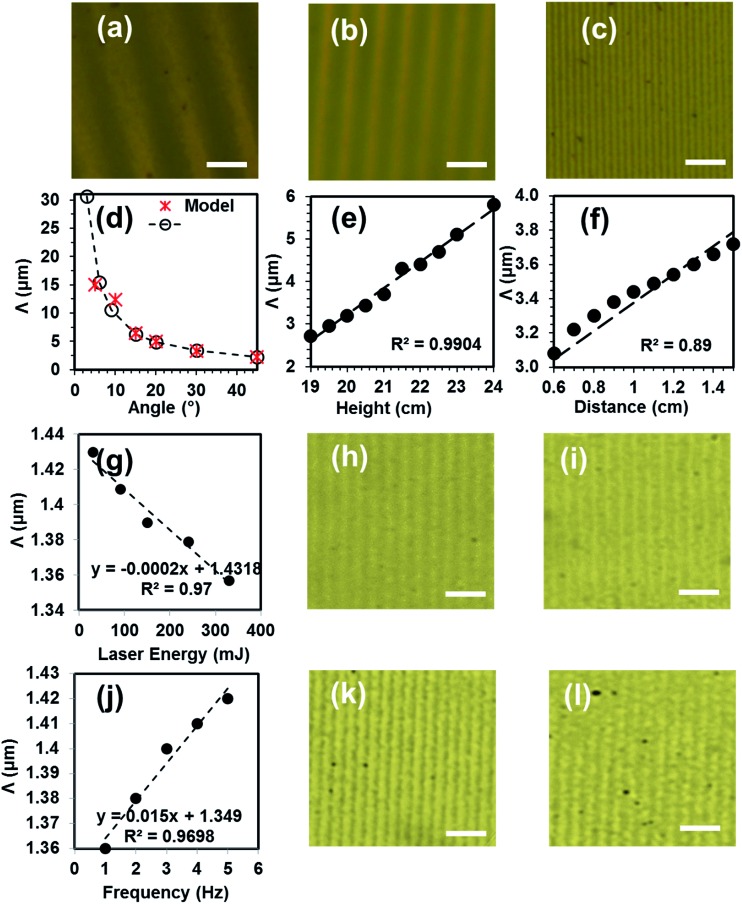
Microscopy images of the Au–Ti surface gratings fabricated by holographic DLIP. (a) Grating with periodicities of 7.7 μm, (b) 3.28 μm, and (c) 960 nm. The effect of changing (d) tilt angle, (e) mirror-sample distance, and (f) laser beam-sample distance on grating periodicity. (g) The effect of changing energy on grating bar width. Microscopy images of 25 nm Au sample produced with (h) energy of 90 mJ and periodicity of 1.4 μm and with (i) energy of 330 mJ and periodicity of 1.3 μm. (j) The effect of altering number of pulses on grating periodicity. Optical microscopy images of grating made with 330 mJ energy at (k) 3 and (l) 4 Hz. Scale bar = 5 μm.

Fig. 2a illustrates the computation geometry and E-field intensity distribution for the focused laser beam interference. The normalized intensity profiles were computed for the tilted observation plane angle variation (*θ* = 30° and 45°). Peak distance decreased with larger tilt angles (Fig. 2b). Fig. 2c and d shows focused beam inference spacing (*Λ*) as a function of tilt angle of observation plane (*θ*) and distance variation (Fig. 3d). As tilt angle of the observation plane increased, the interference spacing shifted to lower values. Similarly, distance was varied to compute interference spacing. As the distance between mirror and observation plane increased, the interference spacing increased linearly. Hence, a 2D simulation was performed for the computation simplicity, reduce computation time with a μm range simulation domain that was smaller than the experiment. In addition, simulations at different wavelengths were performed. Fig. 3e is showing interference nodes as a function of wavelength. As the optical wavelength is increased the grating spacing is also increased.

### Diffraction grating and 2D patterns fabrication

An Nd:YAG laser beam (*λ* = 1064 nm, 3.5 ns pulse) with a single pulse energy on the order of 330 mJ was used. Au–Ti film (Au 10 nm, Ti 4 nm) was patterned through controlling the tilt angle with respect to the surface plane of the mirror to create well-ordered gratings with different periodic spacings at each angle. Fig. 3a–c shows the microscopy images of Au–Ti surface gratings with varying periodicity based on the tilt angle (*θ*). The periodicity of the grating was 7.7 μm when the tilt angle was 6° (Fig. 3a); and as it increased to 15°, the periodicity decreased to 3.28 μm (Fig. 3b). This approach allowed for creating highly-controllable gratings having a periodicity of ∼980 nm at a tilt angle of 45° (Fig. 3c).

The effects of the laser writing parameters on the grating formation were experimentally studied (Fig. 3d–f). The tilt angle was varied between 0° to 45°. Since the beam was not collimated, reducing the two parameter height, *h* (lens – recording medium) and distance, *d* (sample – object) also contributed to decreasing the grating spacing (Fig. 3e and f). The tilt angle variation had the highest impact on decreasing the grating spacing, and by using the three parameters together, the gratings were controllably fabricated and optimized.

Further studies were done on 25 nm Au coated samples. The beam energy was varied from 30 mJ to 330 mJ. It was found that by increasing the energy, the width of grating bars were decreased which is due to more material ablation caused by more energy delivered to the substrate (Fig. 3g). The optical microscopy images of structures made by 330 mJ are shown in Fig. 3h and i. Additionally, the number of consecutive pulses at same region was altered from 1 to 4. By increasing the number of pulses, negligible changes in grating spacing were observed. However, it was found that the width of periodic grating bars is reduced, possibly again due to more energy deposition and further ablation. The experiments were repeated several times to show that the fabrication technique was repeatable (the experiments are detailed in ESI†).

**Fig. 4:**
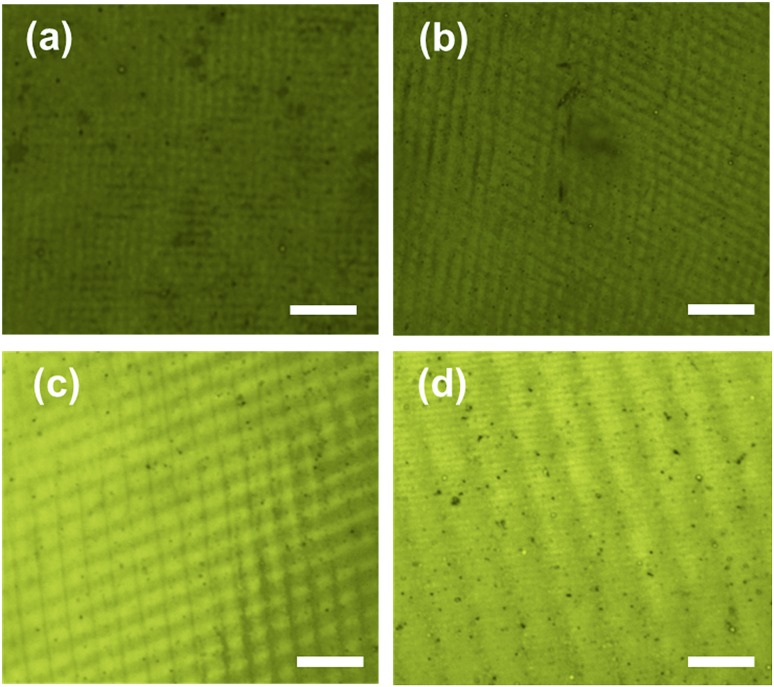
2D Au square arrays fabricated by holographic DLIP. Arrays of 25 nm thick (a) squares (640 nm × 640 nm) and (b) parallelograms (2.4 μm × 1.8 μm). Arrays of 40 nm thick (c) elongated squares (2.98 μm × 3.04 μm) and (d) parallelograms (5.8 μm × 950 nm). Scale bar = 10 μm.

2D periodic Au nanostructures were also fabricated by exposing the recording medium to multiple beam pulses. After the first exposure (1D grating formation), the sample was rotated 90° (Fig. 1) and subsequently exposed to laser light to produce 2D grating periodicity. By varying the rotation/tilt angles, structures with square and rectangular (parallelogram) arrays were produced. Fig. 4 illustrates the fabricated structures in 25–40 nm thick Au films. Fig. 4a shows 25 nm 2D pattern of square arrays (90° rotation) with dimension of each square near 640 nm × 640 nm produced at a tilt angle of 30°. This smallest feature size and grating distance was achieved by optimizing the three parameters.^2,21^ Fig. 4c and d show a 40 nm thick array of squares (2.98 μm × 3.04 μm) and rectangles (5.8 μm × 950 nm).

### Optical characterization of the gratings

**Fig. 5:**
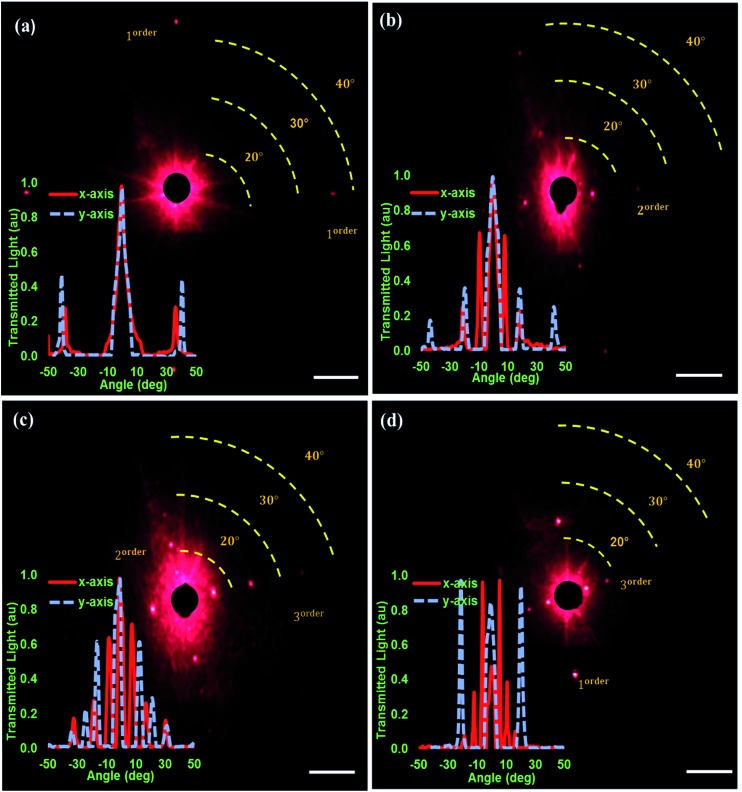
Diffraction of monochromatic light from 2D nanopattern arrays. Light diffraction from (a) squares (640 nm × 640 nm), (b) rectangles (2.4 μm × 1.8 μm) (c) squares (2.98 μm × 3.04 μm), and (d) rectangles (parallelograms) (5.8 μm × 950 nm). Angle-resolved measurements of diffraction intensity for the diffraction patterns shown in (a–d) respectively. Scale bar = 10 μm.

The diffraction of light from 2D arrays was analyzed by normally illuminating the periodic samples with a red laser beam (*λ* = 650 nm) and observing the transmitted light on a perpendicular flat screen. Upon illumination with laser light, the square pattern (640 nm × 640 nm) produced a diffraction pattern consisting of four first order spots, with a distribution along the horizontal and vertical axes (Fig. 5a). The angular distribution of the spots was measured to be ∼40° with respect to the undiffracted light source (zero order in the centre). The optical measurements of diffraction were consistent with four spots locations on horizontal and vertical axes. Gratings having small periodicities resulted in first order diffraction only and large diffraction angles on both axes. The diffraction from a rectangular (parallelogram like) array (2.4 μm × 1.8 μm) produced horizontal axis spots at 10° and 24° for first and second orders, while vertical axis spots were at 23° and 39° for the first and second orders, respectively (Fig. 5b).

An increase in the array periodicity size decreases the diffraction spot angles; however, it was also associated with an increase in the number of diffraction orders (Fig. 5c and d). The diffraction patterns produced for the elongated square (2.98 μm × 3.04 μm) array sample showed three orders (10°, 23°, and 33° horizontally; and at 9°, 20°, and 27° vertically) along each axis. Finally, the interesting results for rectangles (parallelograms) (5.8 μm × 950 nm) in Fig. 5d show that by having none uniform periodicities (in the two axis) customised diffractions can be produced. The results also show that change in the recording medium thickness is a crucial factor for the required diffraction efficiency of optical applications. As the Au thickness of patterns increased from 25 nm (Fig. 5a and b) to 40 nm (Fig. 5c and d), the intensity of the light diffraction spots also increased.^21^ Therefore, both the grating spacing and the thickness of the material influenced the characteristics of light diffraction.

**Fig. 6:**
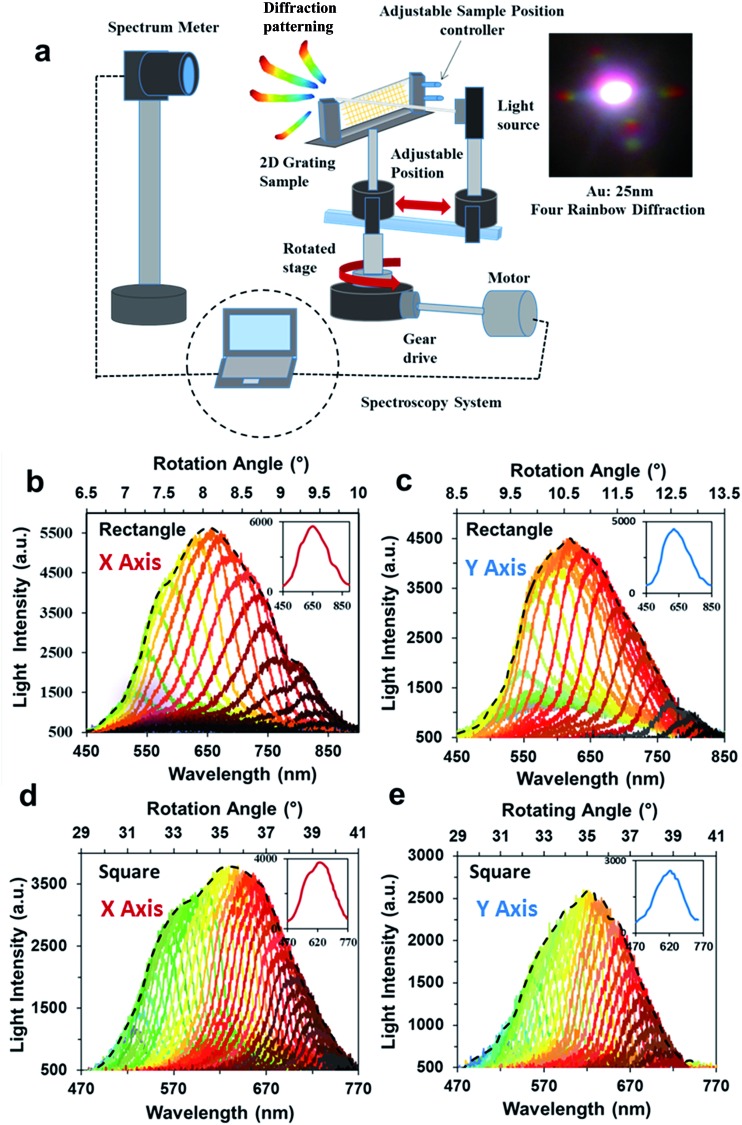
Angle-resolved measurements of the diffraction gratings fabricated *via* holographic DLIP. (a) The spectroscopy system contains a white light beam that passes through a 2D metal film sample on a motorized stage. Transmission spectra of 25 nm thick elongated-rectangular array (2.4 μm × 1.8 μm) (b) *x* axis and (c) *y* axis. Transmission spectra of square array (640 nm × 640 nm) (d) *x* axis and (e) *y* axis.

Angle-resolved measurements were used to analyze the diffraction efficiency of 2D gratings using a halogen light source (Ocean Optics HL-2000). The transmission from the square and rectangular 2D structures generated four visible rainbow patterns. These 25 nm thick Au patterns were analysed in the horizontal and vertical axes. The optical light intensity measurements were conducted on each sample perpendicularly placed 13.5 cm away from the light source. The light diffraction from the 2D samples was spectroscopically analyzed using a motorized rotation stage (Fig. 6a). The stage rotated from –90° to 90° with 1° step increments. This measurement was repeated for each axis to record the distribution of the rainbow diffraction pattern.

Fig. 6b shows angle-resolved measurements of rectangular arrays from 6.5° to 10° from the normal within a spectral range from 450 to 900 nm. The rectangular structure diffracted light at wide bandwidths of 450 nm and 400 nm on horizontal and vertical axes, respectively (Fig. 6b and c).

Fig. 6c shows the vertical axis wavelength spectrum, covering a range from 8.5° to 13.5°. The transmitted light intensity distribution of diffracted light through a square grating in both axes is less than elongated-rectangular arrays. Fig. 6d shows the diffraction of light through a square array measured from 29° to 41°. The highest transmitted light intensity in the *x* axis (∼3900 a.u.) was from 580 nm to 640 nm. However, in vertical axis shown in Fig. 6e, the wavelength range was from 470 nm to 740 nm from 29.5° to 39.5°. The maximum light intensity of the vertical axis was ∼2600 a.u. The transmitted light intensity for the square array was ∼3900 a.u. and ∼2600 a.u. on the horizontal and vertical axes, respectively. The square pattern bandwidth was 300 nm on the horizontal axis and 260 nm on the vertical axis.

## Conclusion

Holographic DLIP has many advantages which allow substantial improvement in the production process, offering low cost, fabrication flexibility and simple setup as compared to other nanofabrication strategies. The present holographic DLIP strategy can generate 1D gratings with single pulse (nanosecond range) and create 2D patterns by multiple pulses to achieve highly controllable nanoscale arrays. Grating spacing was controlled by varying the tilt angle with respect to the object surface plane. In addition, adjusting the distances between the laser-source-mirror also enhanced the control over the grating periodicities. Controlling these parameters allowed the creation of accurate 2D holographic nanostructures. The fabricated Au structures were 2D square and rectangular arrays having thicknesses of 25 nm and 40 nm. Angular-resolved measurements were used to investigate the diffraction characteristics of the 2D gratings produced. Increase in the grating distance also increased the number of visible diffraction orders. A variety of grating shapes, periodicities and the tailored optical effects were rationally designed and characterized. Additionally, the diffracted light efficiency was observed to increase with the Au film thicknesses. The fabricated 1/2D holographic arrays could have applications in biosensors, security devices, and printable optical devices.
