# Supplementary material for: Holographic direct pulsed laser writing of two-dimensional nanostructures
Source: RSC Adv. 2016 Nov 22;6(112):111269–75. doi: 10.1039/c6ra22241b (PMC5171911; doi:10.1039/c6ra22241b)
Supplement: Supplementary file 1 [file RA-006-C6RA22241B-s001.pdf]

Electronic Supplementary Material (ESI) for RSC Advances.
This journal is © The Royal Society of Chemistry 2016

Supplementary material

Holographic Direct Pulsed Laser Writing of Two-Dimensional Nanostructures

Bader AlQattan[1], Haider Butt[1,*], Aydin Sabouri[1], Ali K. Yetisen,[2,3] Rajib Ahmed[1], Nasim Mahmoodi[1]

[1]Nanotechnology Laboratory, School of Engineering, University of Birmingham, Birmingham B15 2TT, UK.

[2]Harvard Medical School and Wellman Center for Photomedicine, Massachusetts General Hospital, 65 Landsdowne Street, Cambridge, MA, 02139, USA

[3]Harvard-MIT Division of Health Sciences and Technology, Massachusetts Institute of Technology, Cambridge, MA, 02139, USA

*E-mail: h.butt@bham.ac.uk. Tel.: +441214158623

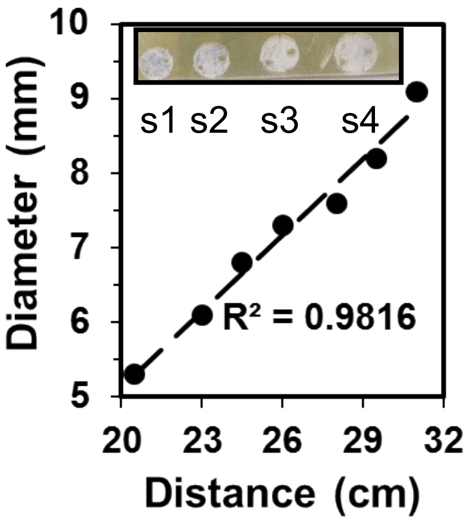

Figure S1: Laser spot size as a function of (h) distance variations. Inset size (diameters): s1 = 7.3 mm, s2 = 7.6 mm, s3 = 8.2 mm, and s4 = 9.1 mm.

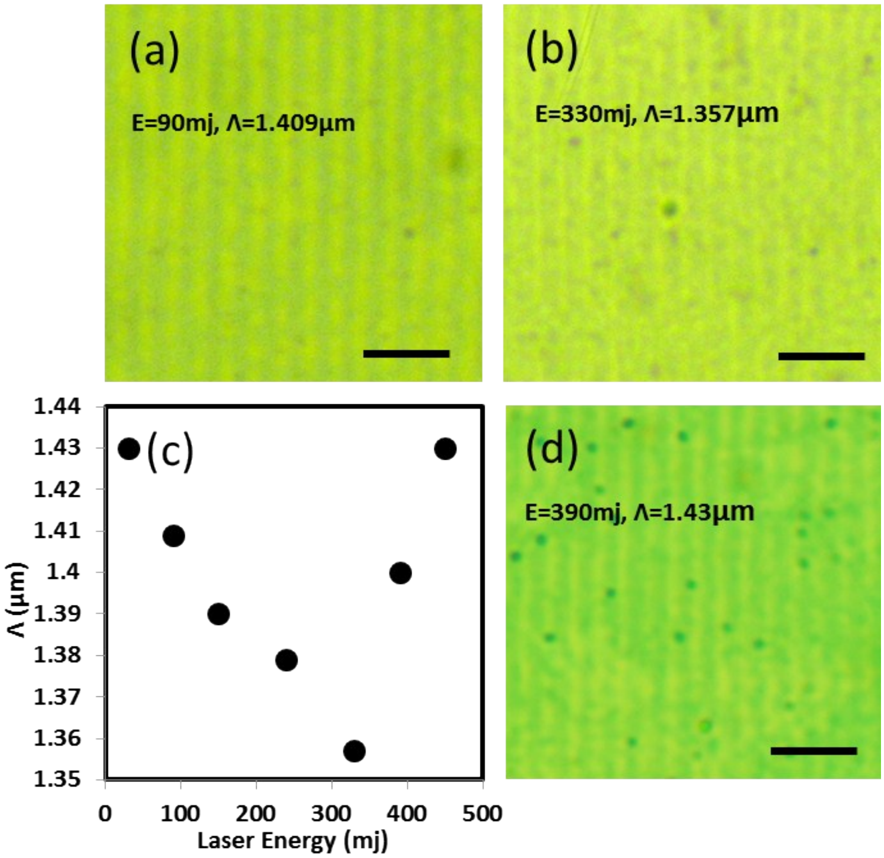

Figure S2: Microscopy images of the Au 25nm thickness surface gratings fabricated by holographic DLIP. (a, b, d) Examples of changing the laser energy on the Au film. (c) The plot for the change of energy effect in the Au film. The graph shows that the effect of laser energy after 330mJ on Au film which increase the removal rate and affect the material quality. Scale bar =5 µm.

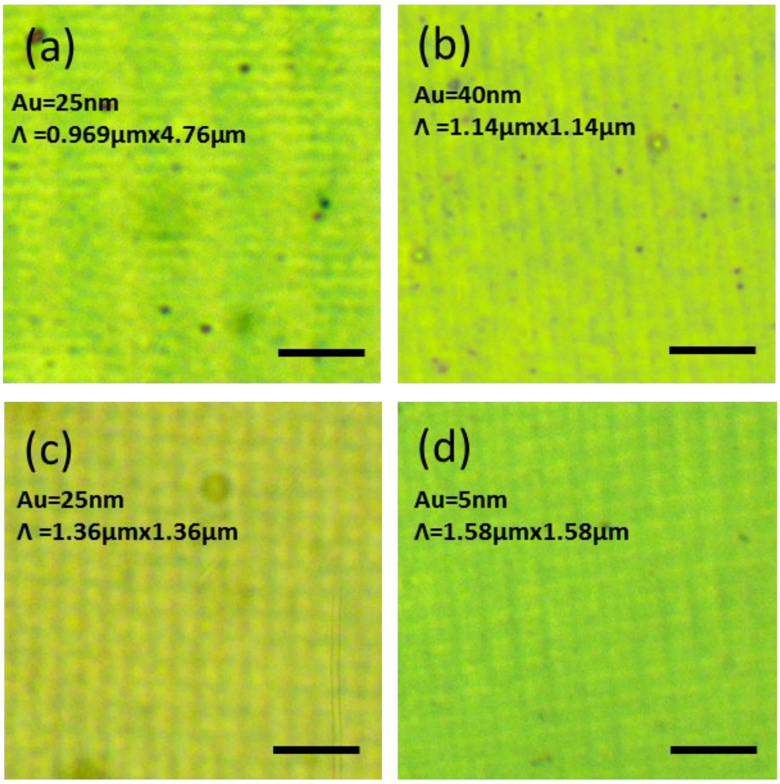

Figure S3: Microscopy images of 2D arrays with different thicknesses fabricated by holographic DLIP. (a) Arrays of 25 nm thick rectangle (0.969 µm ×4.76 µm) and (b) Arrays of 40 nm thick square (1.14µm × 1.14 µm), (c) Arrays of Au 25 nm thick squares (1.36 µm × 1.36µm) and (d) Arrays of 5 nm thick square (1.58 µm × 1.528 nm). Scale bar = 10 µm.

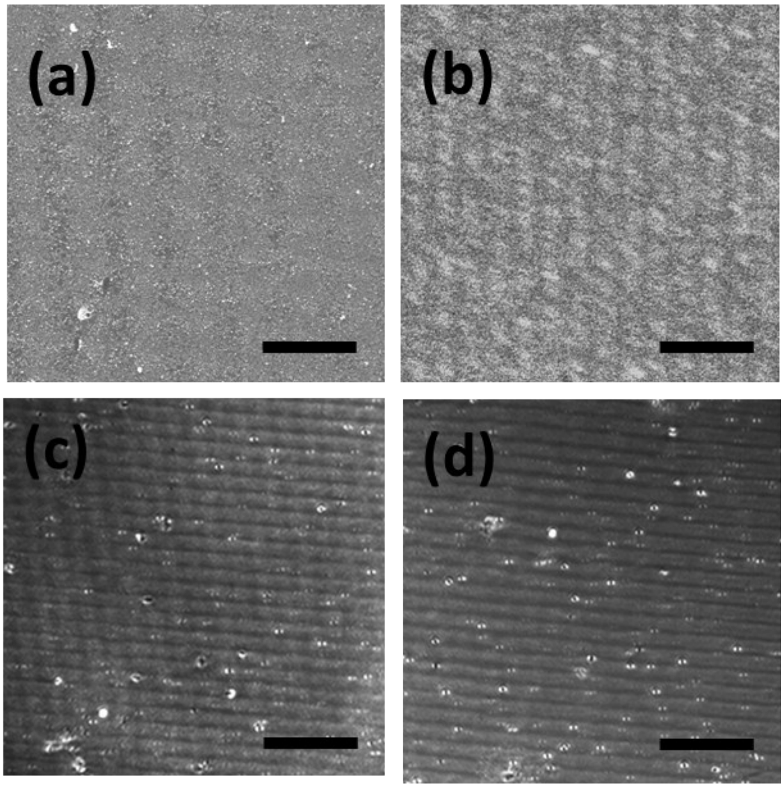

Figure S4: SEM images for 25 nm thick Au surface grating with spacing of (a) 2.4 µm × 1.8 µm and (b) 2.4 µm. Alicona images for (c) 2.4 µm × 1.8 µm and (d) 1.8 µm periodic sample. The scale bars are (a)8µm, (b) 5µm, and (c-d) 12 µm, respectively.

Repeatability

Additional experiments were performed to show the repeatability of this fabrication technique. Figure S6 below shows the gratings made with similar machining parameters on different samples which results in having identical structures. Several Au samples with thickness of 19 nm were ablated by a 150 mJ laser pulse, resulting in a 1.19 µm grating spacing each time (Figure S6a-b). The same exercise was also repeated with samples of 25nm thickness, resulting in a grating of 1.37µm each time (Figure S6c-d).

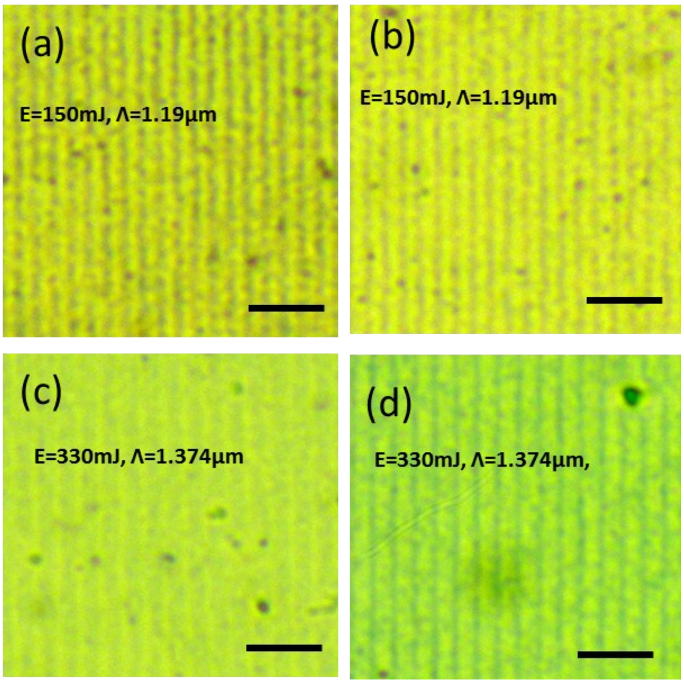

Figure S5: Microscopy images of (a-b) 19 nm Au surface gratings fabricated by holographic DLIP with energy of 150 mJ. (c-d) 25 nm Au surface grating with energy of 330 mJ with spacing of 1.37 µm. Scale bar =5 µm.

We used the following parameter for producing the 1D gratings: 19 nm thickness, 150 mJ laser energy, 21 cm laser beam-sample distance (h), $\Theta=25^{o}$angle, 0.79 cm mirror-sample distance (d) and 1Hz frequency (f) to achieve periodicity of Λ =1.19 µm. We made the two samples in figure S6(a-b) on different days and we get the same periodicity. Similarly, figure S6(c-d) are made with: h = 21.5 cm, Θ = 20°, d=1.8cm, f =1Hz to make grating spacing Λ=1.357µm in both samples.
